# Supplementary material for: Evolution of a distinct chromatin regulatory landscape in brown algae
Source: Nat Ecol Evol. 2026 Mar 27;10(4):779–93. doi: 10.1038/s41559-026-03031-3 (PMC13076208; doi:10.1038/s41559-026-03031-3)
Supplement: Supplementary file 1 — Supplementary Figs. 1–5. [file 41559_2026_3031_MOESM1_ESM.pdf]

nature ecology & evolution

Supplementary information

https://doi.org/10.1038/s41559-026-03031-3

Evolution of a distinct chromatin regulatory landscape in brown algae

In the format provided by the authors and unedited

Supplementary Information

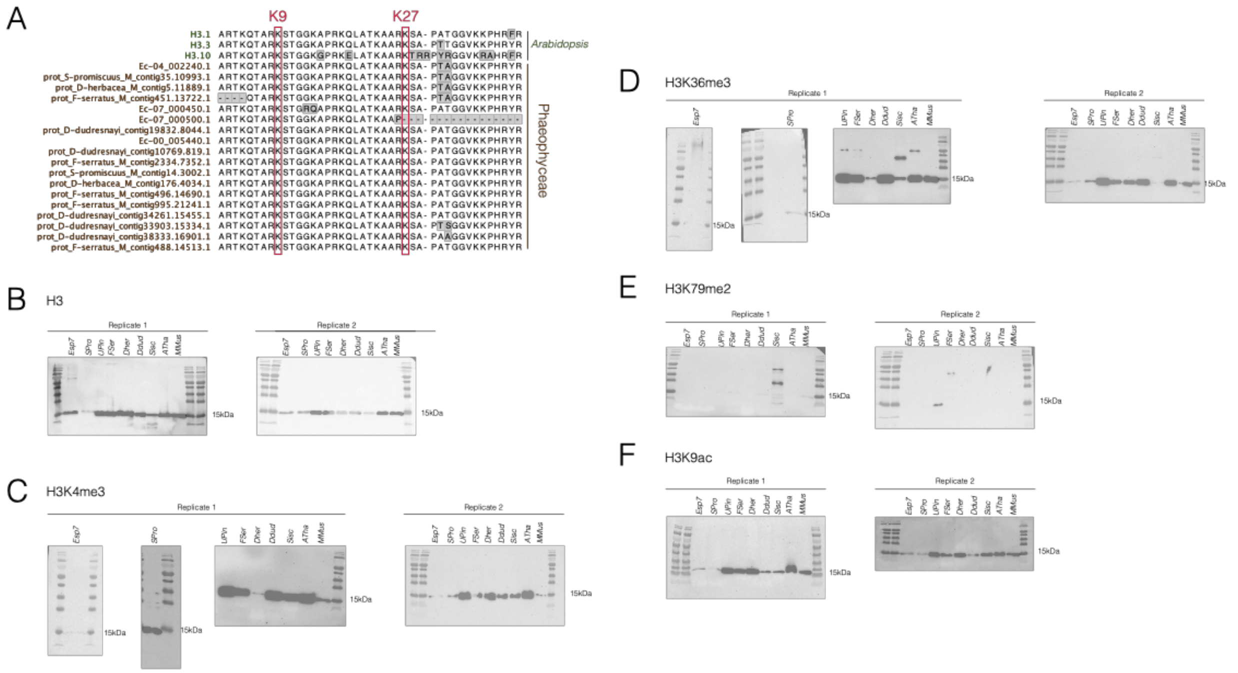

Supplementary Figure 1: (A) Alignment of the N-terminal tail of brown algal histone H3 sequences alongside histone H3.1, H3.3 and the atypical H3K27me3-immune variant H3.10 from *Arabidopsis*. (B-F) Uncropped Western blots of selected hPTMs across species and model organisms such as *Arabidopsis thaliana* (*ATha*) and *Mus musculus* (*MMus*).

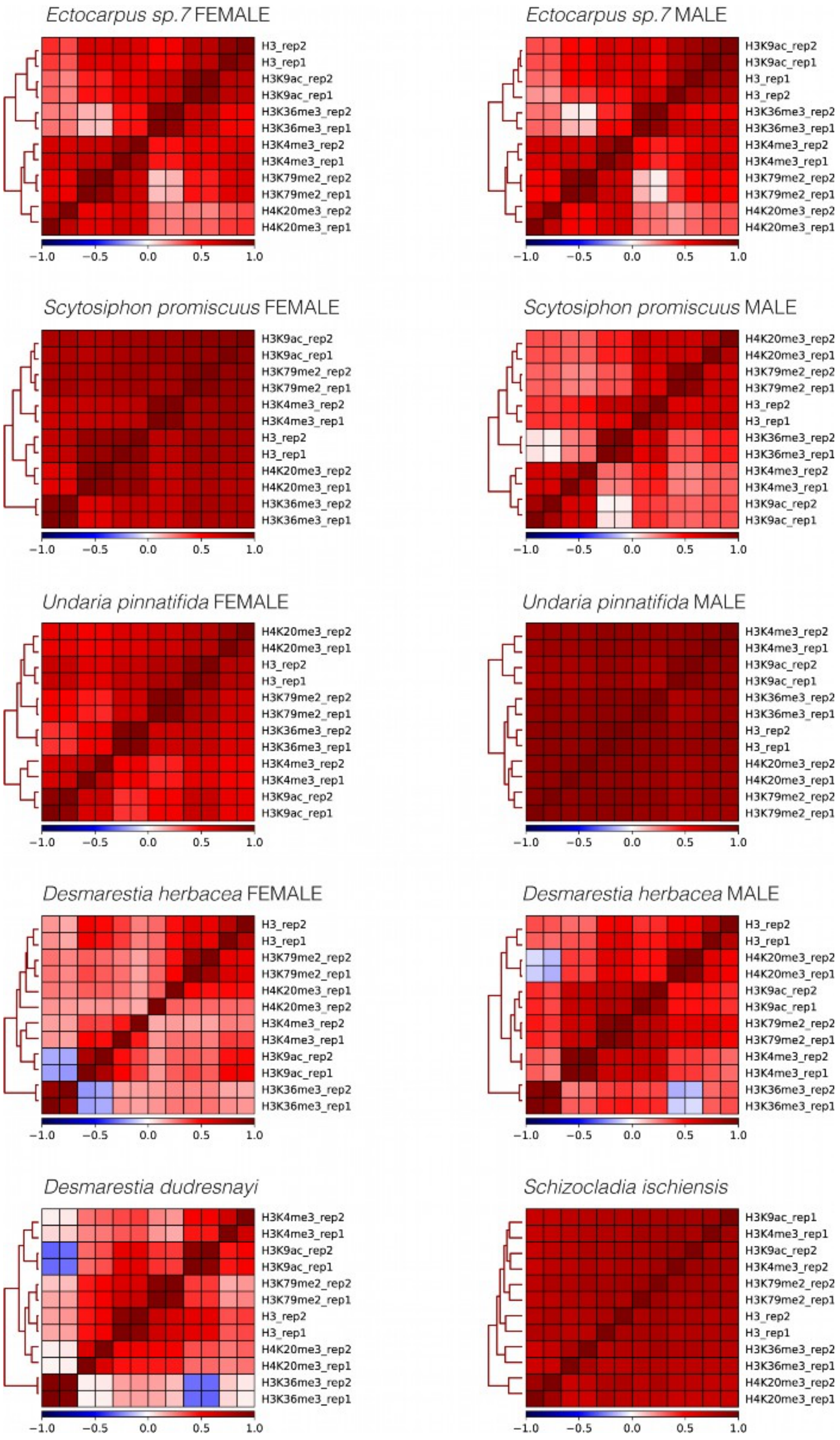

Supplementary Figure 2: Spearman correlation matrix of the ChIP-seq datasets for each species.

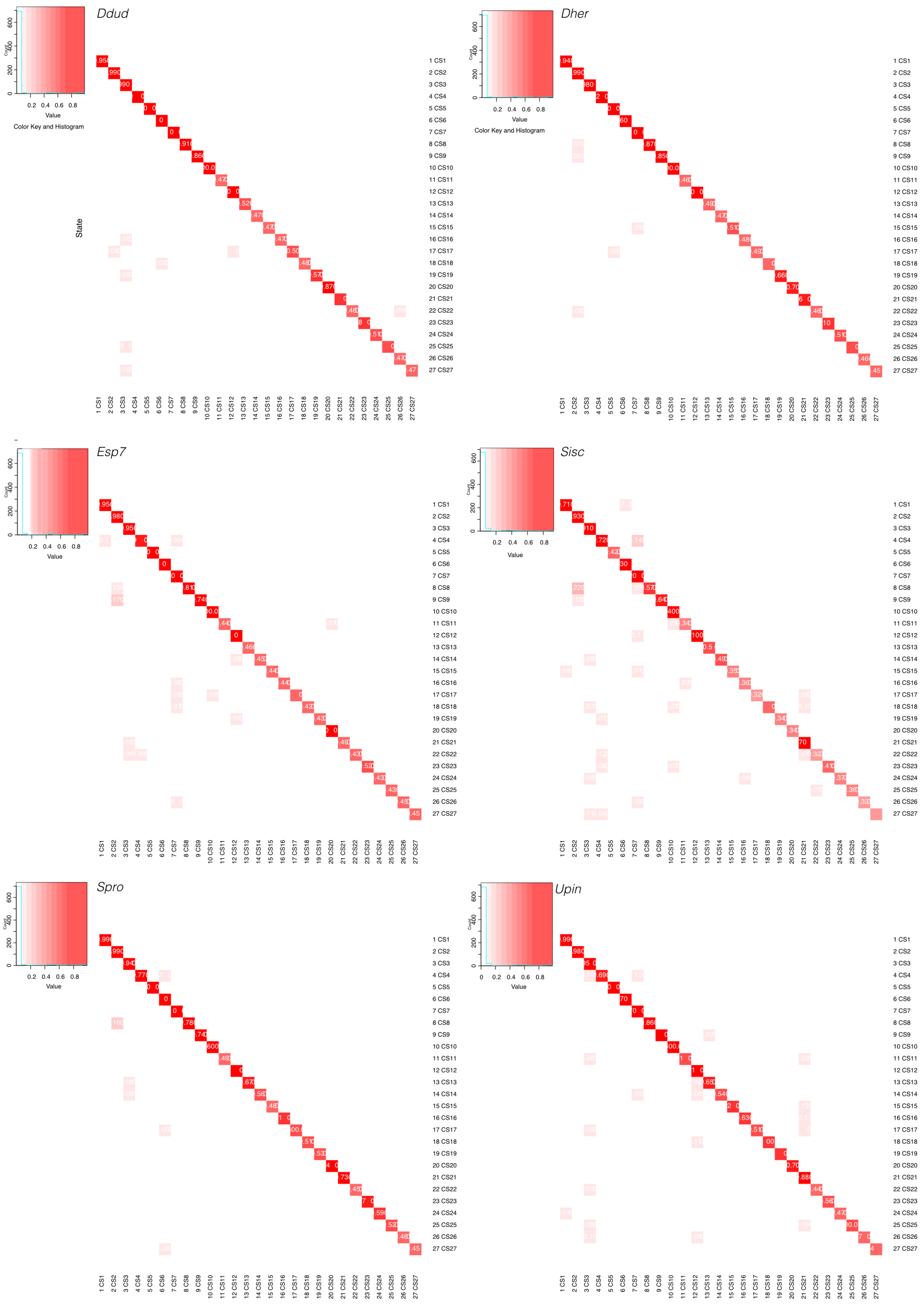

Supplementary Figure 3: Transition probability matrices for the hiHMM chromatin state model. Each panel shows the transition matrix learned by the hiHMM model for a given species. Rows represent the probability of transition

from a chromatin state and columns represent the probability of transitioning to the next state. Warmer color correspond to higher transition probabilities. Strong diagonal elements suggest that states tend to be spatially continuous. Off-diagonal enrichments highlight preferred transition between specific chromatin states. These matrices show how chromatin states are organised along the genome with specific dynamics for each species.

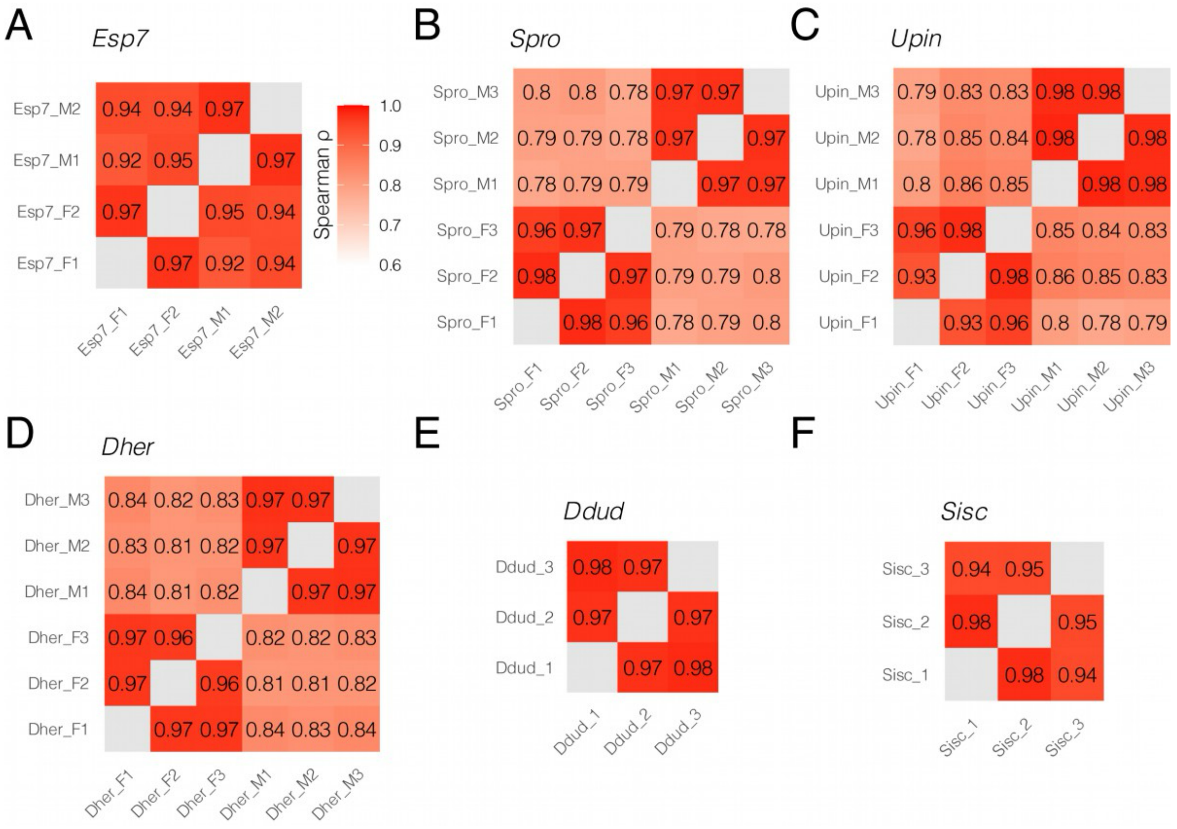

Supplementary Figure 4: Correlation matrices between RNAseq samples used in this study. (**A**) *Ectocarpus* (from [8]), (**B**) *Scytosiphon promiscuus*, (**C**) *Undaria pinnatifida*, (**D**) *Desmarestia herbacea*, (**E**) *Desmarestia dudresnayi*, and (**F**) *Schizocladia ischiensis*. Male and female samples are indicated by suffix "_M" or "_F", respectively. Correlation is measured using Spearman's ρ.

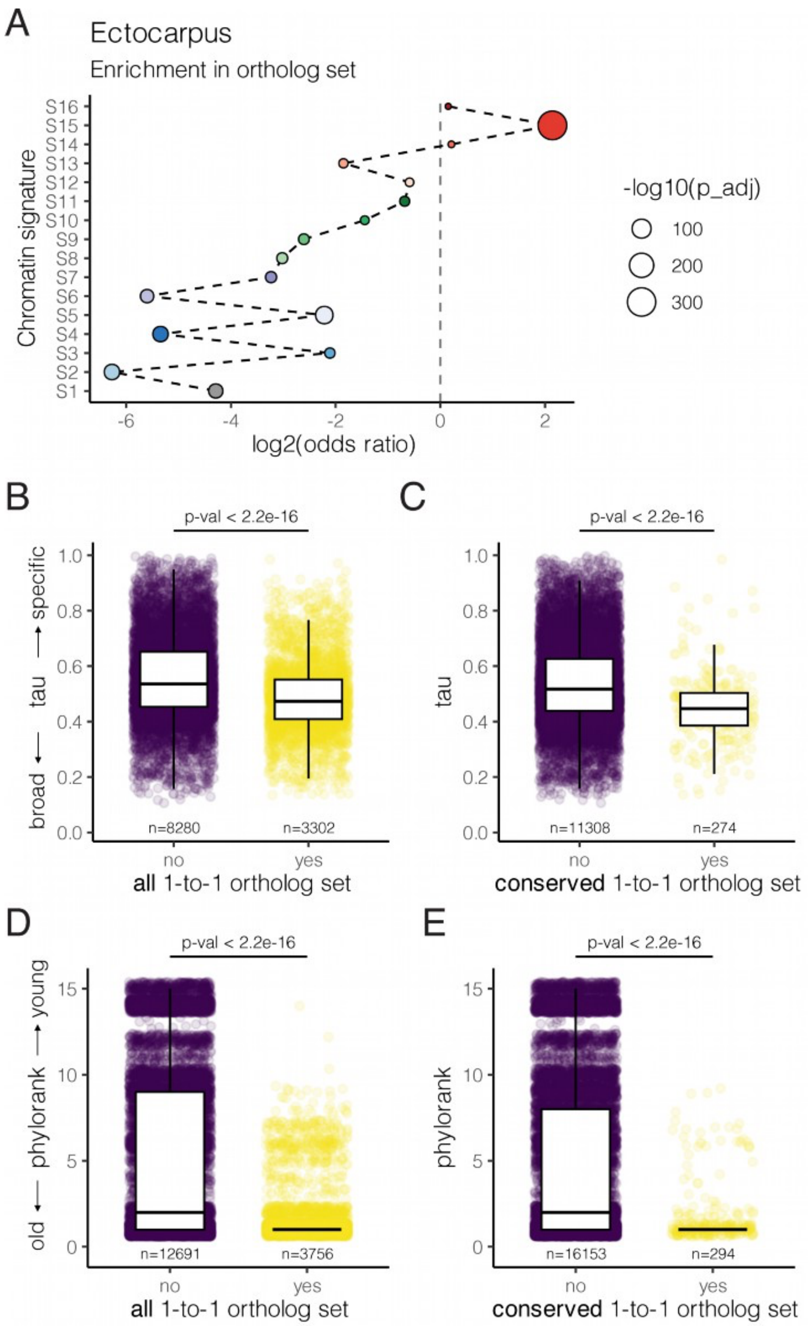

Supplementary Figure 5: Expression and evolutionary profile of genes in the one-to-one ortholog set compared to all other genes in *Ectocarpus*. (**A**) Chromatin signatures enriched in the one-to-one ortholog set. The Benjamini-Hochberg Procedure was used to adjust the p-values computed from Fisher's exact test. (**B**) Distribution of expression specificity score (tau) in all genes on the one-to-one ortholog set and (**C**) in a subset of one-to-one orthologs with conserved chromatin signature across all species. (**D**) Distribution of gene age (phylorank) in all genes on the one-to-one ortholog set and (**E**) in a subset of one-to-one orthologs with conserved chromatin signature across all species. Related to Fig. 3A. The unpaired two-tailed Wilcoxon test is used to compute the p-values in (**B**-**E**).
